# Misincorporation of Galactose by Chondroitin Synthase of *Escherichia coli* K4: From Traces to Synthesis of Chondbiuronan, a Novel Chondroitin-Like Polysaccharide

**DOI:** 10.3390/biom10121667

**Published:** 2020-12-12

**Authors:** Mélanie Leroux, Julie Michaud, Eric Bayma, Sylvie Armand, Sophie Drouillard, Bernard Priem

**Affiliations:** 1 HTL Biotechnology, 35133 Javene, France; mleroux@htlbiotech.com; 2 CNRS, CERMAV, University Grenoble Alpes, 38000 Grenoble, France; a.julie.michaud@gmail.com (J.M.); eric.bayma@cermav.cnrs.fr (E.B.); Sylvie.Armand@cermav.cnrs.fr (S.A.); sophie.drouillard@cermav.cnrs.fr (S.D.)

**Keywords:** chondroitin, K4 chondroitin synthase, NDP-sugar misincorporation

## Abstract

Chondroitin synthase KfoC is a bifunctional enzyme which polymerizes the capsular chondroitin backbone of *Escherichia coli* K4, composed of repeated β3N-acetylgalactosamine (GalNAc)-β4-glucuronic acid (GlcA) units. Sugar donors UDP-GalNAc and UDP-GlcA are the natural precursors of bacterial chondroitin synthesis. We have expressed KfoC in a recombinant strain of *Escherichia coli* deprived of 4-epimerase activity, thus incapable of supplying UDP-GalNAc in the bacterial cytoplasm. The strain was also co-expressing mammal galactose β-glucuronyltransferase, providing glucuronyl-lactose from exogenously added lactose, serving as a primer of polymerization. We show by the mean of NMR analyses that in those conditions, KfoC incorporates galactose, forming a chondroitin-like polymer composed of the repeated β3-galactose (Gal)-β4-glucuronic acid units. We also show that when UDP-GlcNAc 4-epimerase KfoA, encoded by the K4-operon, was co-expressed and produced UDP-GalNAc, a small proportion of galactose was still incorporated into the growing chain of chondroitin.

## 1. Introduction

Bacterial chondroitin is a natural capsular component of some pathogenic strains such as the Gram negative *Escherichia coli* K4 [1], *Pasteurella multocida* type F [2,3], and *Avibacterium paragallinarium* [4]. Chondroitin belongs to the glycosaminoglycan (GAG) family which is found in animal extracellular matrices. Unlike in bacteria, animal chondroitin is normally sulfated and several types of chondroitin sulfate have been reported, depending of sulfation patterns [5]. There is a growing interest in the use of bacterial GAG synthases at the academic and industrial levels in order to produce high yield, structurally well-defined and animal contaminant free products. Synthesis can be achieved either chemo-enzymatically using purified enzymes, or through their expression in suitable, harmless, well known recombinant bacteria. For those purposes, it is critical to characterize and understand the behaviour of available enzymes.

Chondroitin synthase (CS) KfoC has been extensively studied and its tri-dimensional structure has been solved [6]. It is composed of a N-terminal domain A1 which catalyses GalNAc transfer, and a C-terminal domain A2 which catalyses GlcA transfer [7]. KfoC is being used in the engineering of bacterial strains to produce chondroitin at industrial scale [8,9]. Several studies addressed catalytic properties and specificity of KfoC regarding nucleotide-sugars and various acceptors [10,11,12]. However, studies have never been conducted regarding its behaviour in presence of UDP-galactose.

Here, we report the property of KfoC to incorporate galactose in the absence of UDP-GalNAc in vivo. The ability of some degrading enzymes to cut [β3Gal-β4GlcA]_n_ polysaccharide we called “chondbiuronan” is also investigated.

## 2. Materials and Methods

### 2.1. Chemicals and Molecular Biology Supplies and Services

Chemicals, unless otherwise indicated, were purchased from Sigma-Aldrich Chimie (Saint-Quentin-Fallavier, France). Unsulfated chondroitin was from HTL Biotechnology (Javené, France). DNA polymerases, DNA modification, and restriction enzymes were purchased from Thermo-Fisher Scientific Inc. (Illkirch, France). Synthetic DNA was purchased from GenScript Biotech (Leiden, The Netherlands). Chondroitin AC lyase from *Flavobacterium heparinum* and hyaluronidase from bovine testis were purchased from Sigma-Aldrich. DNA sequencing was performed by Eurofins Genomics (Ebersberg, Germany GmbH). Recombinant DNA was isolated using miniprep columns (Qiagen Inc., Valencia, CA, USA). The *E. coli* strain Top10 was used for plasmid construction and strain K-12 for recombinant polysaccharide production in bioreactor.

### 2.2. Bacterial Strains and Recombinant Vectors

The *E. coli* DJ strain (i.e., strain DH1 *lacA lacZ wcaJ*), as well as plasmid constructions pBBR-glcATP-kfiD and pBS-kfoC have previously been used in our laboratory to produce heparosan and chondroitin [13,14]. DNA encoding for UDP-GlcNAc 4-epimerase of *E. coli* K4, KfoA [15] was cloned in Sac1-Sma1 sites of pBAD33 to obtain pBAD-kfoA. Engineered strains constructed in this study are summarized in Supporting information, Table S1.

### 2.3. Culture Media and Culture Conditions for the Production of Polysaccharides

Cultures were carried out in 0.5 L reactors (Infors HT Multifors, Bottmingen, Switzerland) containing 0.2 L of mineral culture medium, as previously described [13]. The high-cell density culture consisted of two phases: (1) an exponential-growth phase at 33 °C, which started with inoculation and lasted until exhaustion of the initially added glucose (17.5 g·L^−1^), and (2) a 72 h fed-batch phase at 28 °C, with 90 mL of 50% glycerol feeding solution, provided at a flow rate of 1.375 mL·h^−1^. isopropyl β-D-1-thiogalactopyranoside (IPTG) (0.2 mM) was added to the culture 3 h after the beginning of the glycerol feed. Lactose acceptor (750 mg) and arabinose (0.5% and 2% *w*/*v* when added) were put in the glycerol feed and supplied continuously. The selecting ampicillin, chloramphenicol, and tetracycline antibiotics were used at 100, 25, and 15 µg·mL^−1^, respectively.

### 2.4. Extraction and Purification of Polysaccharides

After 72 h of fed-batch, the culture was centrifuged (10,000× *g*, 30 min) and the supernatant replaced by distilled water. The cells were then autoclaved at 105 °C for 10 min to be disrupted. The supernatant—designated as the “total extract”—was collected after centrifugation (10,000× *g*, 30 min). IR120 H+ Amberlite resin was used to lower its pH to 3.5 and precipitate proteins. This precipitate was then removed from the solution by centrifugation (10,000× *g*, 30 min). The pH was adjusted to 7 using NaOH, and 3 volumes of ice-cold 95% ethanol were added to the total extract to precipitate the polymer. Finally, the precipitated polysaccharide was isolated using centrifugation (10,000× *g*, 30 min, 4 °C). The pellet was then dissolved in distilled water. Pure chondroitin or chondbiuronan was obtained by anion-exchange chromatography on a MonoQ (HR16/10, GE Healthcare, Uppsala, Sweden) column run on an NGC medium-pressure chromatography system (Bio-Rad, Hercules, CA, USA). The column was washed with 20 mL of 10 mM Tris HCl pH 7.6 buffer, then a linear gradient of 50 mL of 0 to 1 M NaCl in the same buffer was applied. The flow rate was 2 mL·min^−1^ and fractions of 4 mL were collected. The presence of GAGs was monitored by UV detection at 210 nm and confirmed using a colorimetric assay [16]. The presence of contaminants was detected at 260 and 280 nm. Polysaccharide containing fractions were pooled and desalted on HighPrep 26/10 (GE Healthcare, Uppsala, Sweden) desalting column in water. The sample was finally lyophilized and stored at −20 °C for downstream analysis.

### 2.5. Enzymatic Degradation

Chondroitin and chondbiuronan (500 μg) were treated by chondroitin AC lyase from *Flavobacterium heparinum* (0.9 mg) in 500 μL of 50 mM Tris-HCl buffer pH 8 at 25 °C. The reactions were followed by monitoring the formed unsaturated reducing ends at 235 nm.

Chondroitin and chondbiuronan (500 μg) were incubated at 37 °C with hyaluronidase from bovine testis (350 μg) in 250 μL of 0.1 M sodium acetate buffer pH 5 containing 0.1 M NaCl. At regular time intervals, the amount of reducing sugars was determined using the ferricyanide method [17].

### 2.6. Carbohydrate Analyses

Chondroitin and chondbiuronan were quantified by colorimetric assay of uronic acid [16]. Average molecular weight and molecular weight distributions were determined using high-performance size-exclusion chromatography (HPSEC) (LC-20AD, Shimadzu, Marne La Vallée, France) with on-line multi-angle light scattering (MALS) (MiniDAWN TREOS, Wyatt Technology Corp. (Santa Barbara, CA, USA)) fitted with a K5 cell and a laser wavelength of 660 nm, a refractive index detector, and a viscometer. Columns (OHPAK SB-G guard column and OHPAK SB 806 HQ column (Shodex, Munich Germany) were eluted with 0.1 M NaNO_3_ containing 0.03% NaN_3_ at 0.5 mL·min^−1^. Solvent and samples were filtered through 0.1 µm and 0.2 µm filter units (Merck-Millipore, Darmstadt, Germany), respectively. The dn/dc determined with a refractometer was of 0.1306 and 0.1293 for chondbiuronan and chondroitin, respectively. ^13^C and ^1^H-NMR spectra were recorded with a BRUKER Avance 400 spectrometer (Ettlingen, Germany) operating at a frequency of 100.618 MHz for ^13^C and 400.13 MHz for ^1^H. Samples were solubilized in D_2_O and analysed at 353 K. Residual signal of the solvent was used as an internal standard: mono-deuterated water (HOD) at 4.25 ppm.

MALDI-TOF(-TOF)-MS: Matrix solution was prepared by dissolving 100 mg of 2,5-dihydroxybenzoic acid (DHB) in 1 mL of a 1:1 solution of water and acetonitrile then 20 µL of N,N-dimethylaniline (DMA) were added to the DHB matrix solution. The analyte (10 mg/mL in water) and DHB/DMA solutions were mixed (1:1) and spotted onto a polished steel MALDI target, then dried at ambient temperature. The MALDI mass analysis were performed in linear negative ion mode with an Autoflex Speed TOF/TOF spectrometer (BrukerDaltonics, Bremen-Lehe, Germany) equipped with a 355 nm Smartbeam II laser.

## 3. Results

### 3.1. Engineering of E. Coli to Produce Chondroitin/Chondbiuronan Lactose

**Figure 1:**
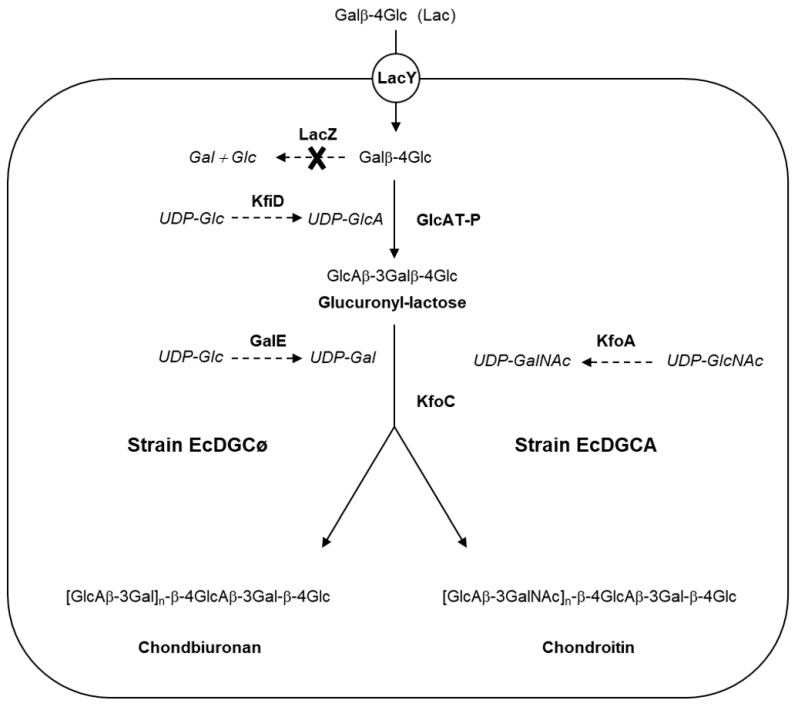
Synthesis of chondbiuronan and chondroitin in engineered *Escherichia coli*. Lactose (Lac) is taken up by lactose permease (LacY) and modified by the action of recombinant mouse β-1,3-glucuronyltransferase (GlcAT-P), leading to glucuronyl-lactose formation, UDP-GlcA being provided by the action of recombinant UDP-Glc dehydrogenase (KfiD) from the *E. coli* strain K5. Recombinant chondroitin synthase KfoC catalyses (i) synthesis of chondbiuronan in strain EcDGCø from UDP-GlcA and UDP-Gal constitutively produced by GalE or (ii) synthesis of chondroitin in strain EcDGCA from UDP-GlcA and UDP-GalNAc produced by recombinant epimerase KfoA. Genotypes: EcDGCø, strain DJ (pBS-kfoC, pBBR-kfiD); EcDGCA, strain DJ (pBS-kfoC, pBBR-kfiD, pBAD-kfoA).

Metabolic engineering of recombinant strains is shown in Figure 1.

The recombinant strain capable of polymerizing exogenously added lactose through the action of GAG synthases was previously described [14]. As a derivative of *E coli* DH1, the strain does not synthesize GalNAc, contrary to *E coli* K4, which expresses KfoA, an UDP-GlcNAc 4-epimerase.

Briefly, genes *lacZ* and *wcaJ* were knocked-out, resulting in the incapacity of the strain to degrade lactose (Lac), or to produce colanic acid which is a GlcA-containing polysaccharide [18]. In addition, recombinant mammal β1,3-glucuronyltransferase GlcAT-P and UDP-Glc dehydrogenase KfiD were expressed, thus allowing the production of glucuronyllactose (GlcA-Lac) upon Lac implementation [19,20]. Genes *kfiD* and *glcAT-P* were both expressed in low copy pBBR, while the gene *kfoC* encoding chondroitin synthase was expressed in high copy pBluescript.

The strain named “EcDGCø” expressing three recombinant genes *kfoC*, *glcAT-P,* and *kfiD* was constructed to examine the capacity of the enzyme KfoC to produce a polysaccharide in absence of UDP-GalNAc.

The strain “EcDGCA” was constructed in order to observe the incorporation of GalNAc while expressing UDP-GlcNAc 4-epimerase KfoA, providing UDP-GalNAc from UDP-GlcNAc epimerisation. Gene *kfoA* was cloned in medium copy pBAD33 carrying the tightly regulated promoter araBAD. Expression of the epimerase was induced by arabinose, independently of the other three recombinant genes induced by IPTG. It was thus possible to modulate the level of epimerase activity without changing the expression of the other recombinant genes. Strain EcDGCA was very similar to strain K-cho that we have described previously, capable of producing recombinant chondroitin from lactose and lactose-furyl [14].

**Figure 2:**
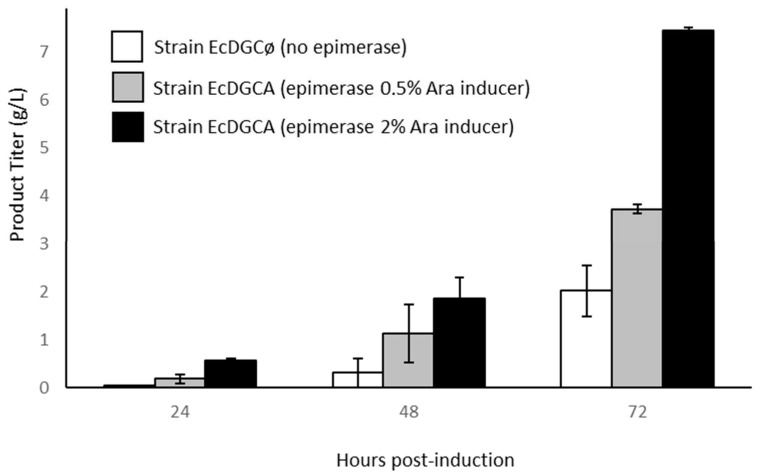
Production of recombinant polysaccharide during growth of engineered strains cultivated in bioreactor. Titer is calculated on the basis of the uronic acid assay. Error bars represent standard deviation of two independent cultures.

Recombinant strains cultures were driven on minimal medium and cultivated for 72 h in fed-bach conditions (Figure 2).

All cultures accumulated an ethanol-precipitated product intracellularly as the synthesis occurred from cytoplasmic lactose and *kps* genes involved in polysaccharide export were absent from K-12 strains [21]. Accordingly, strain EcDGCA accumulated several g·L^−1^ of chondroitin at the end of the culture. Interestingly enough, it could be observed that the rate of production increased with epimerase induction controlled by the addition of arabinose, suggesting that it was a limiting parameter of chondroitin synthesis. We can reasonably hypothesize that the epimerase induction level impacts the cytoplasmic concentration of UDP-GalNAc, probably limiting in the polymerization reaction. Strain EcDGCø culture lacking epimerase slightly accumulates a lower amount of unknown polysaccharide (compound **1**). However, this compound was only noticeable after 2 days of glycerol feeding.

### 3.2. Structural Identification of Unknown Polymer Formed by KfoC in Absence of GalNAc

Compound **1** produced by strain EcDGCø as well as chondroitin produced by EcDGCA were purified and subjected to various analyses. The HPSEC-MALS analysis of compound **1** revealed a very low molecular weight, compared to chondroitin samples (Table 1).

**Figure 3:**
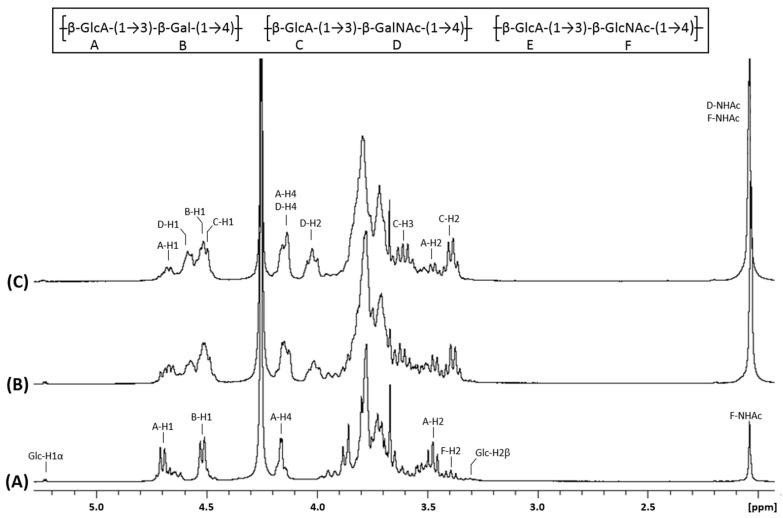
^1^H NMR spectra of chondbiuronan (**A**) and chondroitin obtained using 0.5% (**B**) and 2% (**C**) arabinose induction. Inset: chemical structure of the polysaccharides.

**Figure 4:**
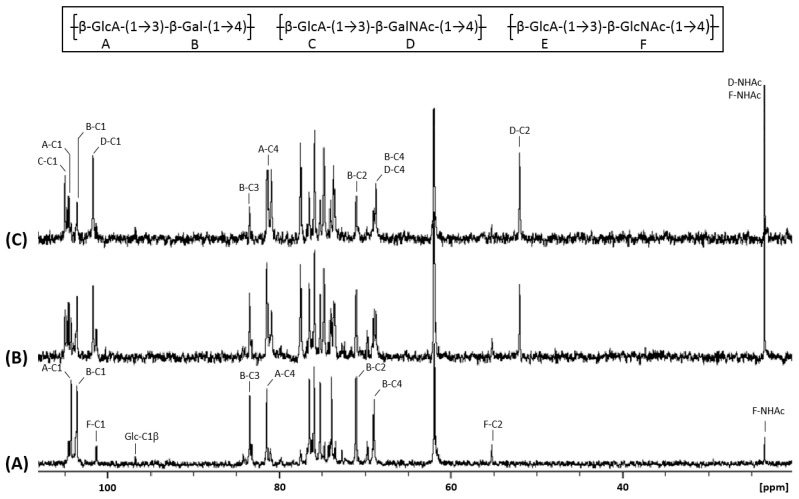
^13^C NMR spectra of chondbiuronan (**A**) and chondroitin obtained using 0.5% (**B**) and 2% (**C**) arabinose induction. Inset: chemical structure of the polysaccharides.

We then acquired the ^1^H NMR (Figure 3A) and ^13^C NMR (Figure 4A) spectra of compound **1**. They showed characteristic signals of glucuronic acid and galactose linked in β1-3 and β1-4 respectively. Indeed, this data proved that KfoC incorporated galactose in absence of UDP-GalNAc, enough to lead to the synthesis of a small chondroitin-like polysaccharide we named “chondbiuronan.”

**Figure 5:**
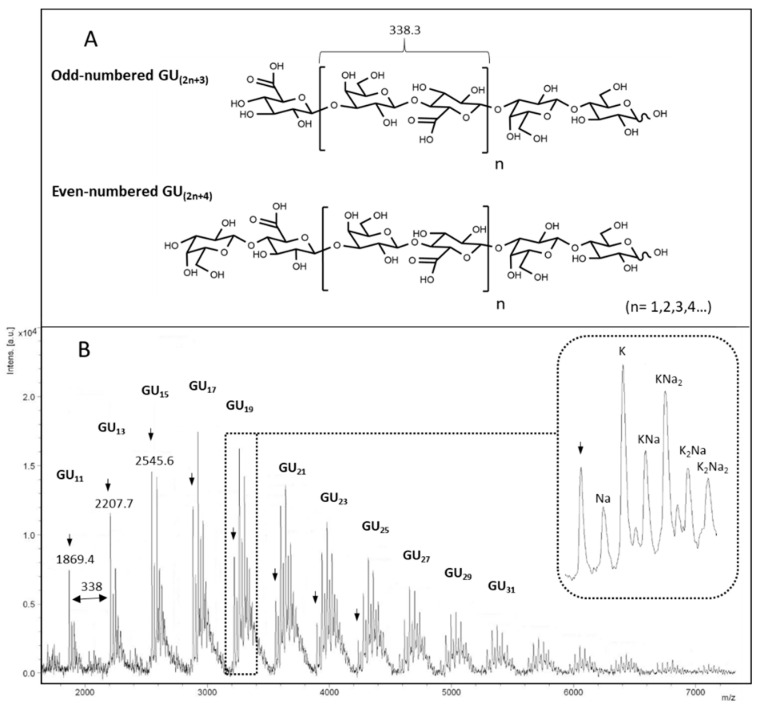
Possible structures of chondbiuronan depending on reducing termini (**A**) and negative-ion linear MALDI mass spectrum of compound **1** (**B**). Acidic [M-H]^-^ peaks are denoted by arrows. Na/H and K/H exchanges are observed increasingly with molecular weight (enlarged peaks).

Considering the very low size of chondbiuronan, we decided to run a mass spectrometry analysis. MALDI-TOF-MS analysis in linear negative mode allowed to obtain several molecular ion clusters separated by 338.3 daltons which correspond to the [Gal-GlcA] motif (Figure 5).

Although this analysis does not reflect the true size repartition of the sample as signal detection decreases with size, we clearly see that main molecular ions belong to odd-numbered polymers, indicating that they contain GlcA at non-reducing termini and lactosyl motif at the reducing termini. The fact that GlcA residues terminate elongating chains suggests that the glucuronyltransferase activity of KfoC was not limiting in the polymerization reaction, and confirms that the GalNAc-T activity was the limiting reaction instead, as suggested before.

### 3.3. Galactose Incorporation Remains in Recombinant Chondroitin

We compared the ^1^H and ^13^C NMR spectra of chondbiuronan (Figure 3A and Figure 4A) to the ones of chondroitin produced by the strain EcDGCA at different induction levels of KfoA; 0.5% (Figure 3B and Figure 4B) and 2% arabinose (Figure 3C and Figure 4C). In addition to the signals found with chondbiuronan, they showed NMR signals that we attributed to N-Acetylgalactosamine (GalNAc) residues as well as glucuronic acid (GlcA) residues linked to GalNAc rather than Gal. The level of incorporated Gal could be calculated according to the ^1^H NMR signals integration: the Gal:GalNAc ratio was of 4:6 and 2:8 at 0.5% and 2% arabinose, respectively, arguing for a competition between Gal and GalNAc incorporation, related to the level of induction of KfoA: the more UDP-GalNAc, the less galactose incorporation.

The chemical shifts of the corresponding protons and carbons were determined using heteronuclear single-quantum correlation experiments (HSQC, Supplementary Figure S1) and correlation spectroscopy (COSY, Supplementary Figure S3) and are reported in Table 2.

### 3.4. N-Acetylglucosamine: Another Misincorporation of KfoC

KfoC is known to incorporate GlcNAc from UDP-GlcNAc in vitro [7]. However, this incorporation has been reported to forbid further polymerization of the growing chain. The NMR experiments of our samples also show the presence of GlcNAc residues in the polysaccharide chains. The F-H3/F-C3 = 3.78/84.21 signals suggest that the third GlcNAc carbon is linked to another GlcA, which highlight the fact that in vivo, the chain elongation continues after the incorporation of a GlcNAc residue. Interestingly, the resulting motif corresponds to the hyaluronan structure. The percentage of GlcNAc incorporated (13%) in the polysaccharide chain was calculated from the ^1^H NMR signals integration (Figure 3A) by comparing the integrals of anomeric protons and protons from the GlcNAc acetyl residue. The chemical shifts have been determined and are reported in the Supplementary Figure S2.

### 3.5. Enzymatic Susceptibility of Chondbiuronan

GAG can be enzymatically depolymerized either by eliminative cleavage with lyases (EC 4.2.2.-) resulting in disaccharides or oligosaccharides with ∆4,5-unsaturated uronic acid residues at their non-reducing end or by hydrolytic cleavage with hydrolases (EC 3.2.1.-).

Chondroitin AC lyase from *Flavobacterium heparinum* (EC 4.2.2.5) cleaves a variety of GAGs, including chondroitin sulfates A (chondroitin 4-sulfate) and C (chondroitin 6-sulfate), as well as the unsulfated chondroitin and hyaluronic acid [22]. Bovine testis hyaluronidase (EC 3.2.1.35) is an endo-β-N-acetyl-D-hexosaminidase that hydrolyses hyaluronan and to a lesser extent sulfated or unsulfated chondroitin [23].

**Figure 6:**
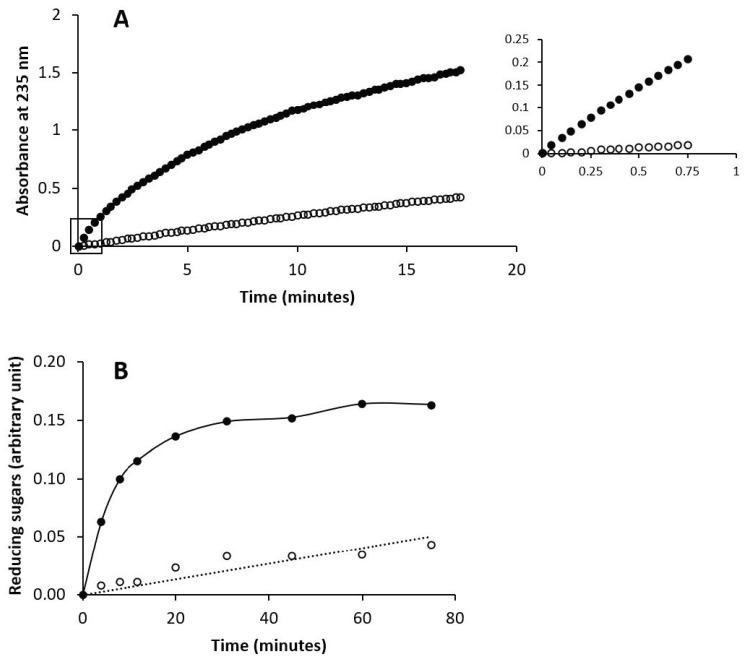
Enzymatic digestion of chondbiuronan and chondroitin by chondroitin AC lyase (**A**) and by hyaluronidase (**B**). The filled or hollow circles represent activity on chondroitin and chondbiuronan respectively. A_235_ is proportional to the concentration of unsaturated non reducing ends formed by the lyase action. Hyaluronidase activity was followed by measuring reducing sugars released from substrates with the ferricyanide method.

Here, the capacity of chondbiuronan to be degraded by these enzymes was evaluated compared to unsulfated chondroitin. As shown in Figure 6, both enzymes could degrade chondbiuronan. Hydrolysis rates of chondbiuronan by chondroitin AC lyase and hyaluronidase were however decreased of about 10-fold and 30-fold respectively, when compared to chondroitin.

## 4. Discussion

Glycosyltransferases are assumed to be very specific enzymes and misincorporation of NDP-sugars is not common in normal circumstances. Nevertheless, the incorporation of non-natural analogues of N-acetamido sugars carrying various N-groups is a well-known approach for carbohydrate labelling or drug design [24,25]. To some extent, KfoC has been found to be able to take up UDP-GlcNAc as a donor, but no further elongation could be observed after that [7]. In this study, we showed that in vivo, elongation after the incorporation of GlcNAc was still possible, leading to a co-polymer composed of hyaluronic acid and chondroitin. To our knowledge, the ability of KfoC to use UDP-Gal as a donor has not been investigated, nor the presence of galactose among chondroitin backbone. The crystal structure of KfoC has been elucidated, but the binding of UDP-Gal in the binding pocket has not been investigated [26].

Here we showed that in vivo, in the absence of UDP-GalNAc, galactose was incorporated by KfoC leading to the synthesis of a chondroitin-like polymer. A similar phenomenon has already been observed with the heparosan synthase PmHS2. In vitro experiments show that PmHS2 incorporates glucose in absence of UDP-GlcNAc, resulting in a new heparosan-like polymer [-4-GlcAβ1-4-Glcα1-]n named hepbiuronan [26]. Following the same rationale, we named the new chondroitin-like polymer: chondbiuronan. Interestingly, the PmHS enzyme used for in vitro hepbiuronic synthesis showed a 10-fold decrease in maximum velocity. Here, comparison of the chondbiuronan and chondroitin synthesis suggests a similar phenomenon occurring with KfoC.

Another work dealing with human α-1,3-GalNAc-T (GTA) involved in blood group reported that the enzyme could bind UDP-Gal but could not efficiently stabilize the complex in a catalytically active conformation [27]. Such a control based on the enzyme mobility does not seem to be very efficient in KfoC. This lack of selectivity may be due to the fact that in the context of recombinant strains, engineered metabolic pathways are different from what they are in wild type strains from which recombinant enzymes are issued, and this can result in enhancing aberrant enzymatic behaviour. In our work, only *kfoA* and *kfoC* genes belonging to the chondroitin operon of K4 have been expressed. It cannot be excluded that in normal circumstances, protein interactions occur, which play a role in enzymatic stability and specificity. Moreover, recombinant *kfoC* gene was cloned in high copy plasmid which supposedly differs from *E. coli* K4 wild type expression. Eventually, we pointed out the fact that galactose could be incorporated in the chondroitin backbone when overexpressing KfoC. The NMR study we have conducted should be of great help to investigate the presence of galactose in bacterial chondroitin produced in other biological systems.

We showed that chondbiuronan could be digested by chondroitin AC lyase and by hyaluronidase. Previously, it has been observed that hepbiuronan could be digested by the bacterial heparin lyase III [26]. These results suggest that the specificity for acting on the hexose-substituted GAGs is relaxed in both classes of bacterial enzymes which are widely used in the industry for quality control of GAGs.

## 5. Conclusions

The fact that chondroitin synthase KfoC could polymerize a GalNAc-free polysaccharide certainly is the most striking result in the present study. The very low molecular weight of the new polysaccharide specie we called chondbiuronan is quite an advantage for chemical coupling and possible use in material coating or drug delivery due to its chondroitin-like structural features and because it could be slowly eliminated by GAG degrading enzymes. The use of lactose derivatives carrying chemical groups such as propargyl, allyl, and furyl groups should be possible as it has been shown that those precursors could be efficiently taken up to produce GAG derivatives in vivo allowing conjugation by click-chemistry [14,28].

## Figures and Tables

**Table 1:** HPSEC-MALS analysis of chondbiuronan and chondroitin in this work. Error is the mean of two independent experiments.

Culture	Mn	Mw	Mw/Mn
EcDGCø + IPTG	6000 ± 1400	8000 ± 2000	1.34 ± 0.01
EcDGCA + IPTG + 0.5% Ara	13,700 ± 1400	24,000 ± 4800	1.79 ± 0.41
EcDGCA + IPTG + 2% Ara	18,600 ± 1800	30,400 ± 3000	1.63 ± 0.01

**Table 2:** ^1^H and ^13^C NMR chemical shifts (δ, ppm) of chondbiuronan (EcDGCø) and chondroitin (EcDGCA) obtained using 0.5% and 2% arabinose induction.

Sugar Residue		1	2	3	4	5	6 (6a,6b)	NHAc (CH3, CO)
Chondbiuronan EcDGCø								
**A**→4)-β−D-GlcA-(1→	^1^H	4.70	3.47	3.65	3.78	3.86	no	
	^13^C	104.24	73.91	75.25	81.43	76.51	175.78	
**B**→3)-β−D-Gal-(1→	^1^H	4.52	3.71	3.79	4.16	3.72	3.79, 3.79	
	^13^C	103.57	71.05	83.42	68.97	75.95	61.94	
Chondroitin EcDGCA								
**A**→4)−β−D-GlcA-(1→	^1^H	4.68	3.47	3.65	3.78	3.85	no	
	^13^C	104.53	74.01	75.25	81.43	76.53	175.78	
**B**→3)−β−D-Gal-(1→	^1^H	4.52	3.71	3.79	4.16	3.72	3.79, 3.79	
	^13^C	103.57	71.03	83.45	68.99	75.95	61.94	
**C**→4)−β−D-GlcA-(1→	^1^H	4.51	3.39	3.61	3.78	3.72	no	
	^13^C	104.99	73.65	74.80	80.93	77.52	175.11	
**D**→3)-β-D-GalNAc-(1→	^1^H	4.58	4.02	3.82	4.14	3.72	3.69, 3.94	2.04
	^13^C	101.71	52.01	81.36	68.76	75.88	62.00	23.47, 175.75

## Supplementary Materials

The following are available online at https://www.mdpi.com/2218-273X/10/12/1667/s1, Figure S1: HSQC spectra of chondbiuronan (A) and chondroitin (B) obtained using 2% arabinose induction. Inset: chemical structure of the polysaccharides, Figure S2: HSQC spectrum of chondbiuronan showing the presence of Glc and GlcNAc incorporated in the polymer. Inset: chemical structure of the polysaccharides, Figure S3: COSY spectra of chondbiuronan (A) and chondroitin (B) obtained using 2% arabinose induction. Inset: chemical structure of the polysaccharides, Table S1: Genes, plasmids and *E. coli* strains used in this study.

## References

[1] Rodriguez M.L., Jann B., Jann K. (1988). Structure and serological characteristics of the capsular K4 antigen of *Escherichia coli* 05:K4:H4, a fructose-containing polysaccharide with a chondroitin backbone. Eur. J. Biochem..

[2] Rimler R.B. (1994). Presumptive identification of *Pasteurella multocida* serogroup A, serogroup D and serogroup F by capsule depolymerization with mucopolysaccharidases. Vet. Rec..

[3] Rimler R.B., Register K.B., Magyar T., Ackermann M.R. (1995). Influence of chondroitinase on indirect hemagglutination titers and phagocytosis of *Pasteurella multocida* serogroups A, D and F. Vet. Microbiol..

[4] Wu J.-R., Chen P.-Y., Shien J.-H., Shyu C.-L., Shieh H.K., Chang F., Chang P.-C. (2010). Analysis of the biosynthesis genes and chemical components of the capsule of *Avibacterium paragallinarum*. Vet. Microbiol..

[5] Truppe W., Kresse H. (1978). Uptake of Proteoglycans and Sulfated Glycosaminoglycans by Cultured Skin Fibroblasts. JBIC J. Biol. Inorg. Chem..

[6] Osawa T., Sugiura N., Shimada H., Hirooka R., Tsuji A., Shirakawa T., Fukuyama K., Kimura M., Kimata K., Kakuta Y. (2009). Crystal structure of chondroitin polymerase from K4. Biochem. Biophys. Res. Commun..

[7] Ninomiya T. (2002). Molecular Cloning and Characterization of Chondroitin Polymerase from *Escherichia coli* strain K4. J. Biol. Chem..

[8] Zanfardino A., Restaino O.F., Notomista E., Cimini D., Schiraldi C., De Rosa M., De Felice M., Varcamonti M. (2010). Isolation of an *Escherichia coli* K4 kfoC mutant over-producing capsular chondroitin. Microb. Cell Factories.

[9] Jin P., Zhang L., Yuan P., Kang Z., Du G., Chen J. (2016). Efficient biosynthesis of polysaccharides chondroitin and heparosan by metabolically engineered *Bacillus subtilis*. Carbohydr. Polym..

[10] Sobhany M., Kakuta Y., Sugiura N., Kimata K., Negishi M. (2012). The structural basis for a coordinated reaction catalysed by a bifunctional glycosyltransferase in chondroitin biosynthesis. J. Biol. Chem..

[11] Sugiura N., Shimokata S., Minamisawa T., Hirabayashi J., Kimata K., Watanabe H. (2008). Sequential synthesis of chondroitin oligosaccharides by immobilized chondroitin polymerase mutants. Glycoconj. J..

[12] Xue J., Jin L., Zhang X., Wang F., Ling P., Sheng J.-Z. (2016). Impact of donor binding on polymerization catalyzed by KfoC by regulating the affinity of enzyme for acceptor. Biochim. Biophys. Acta (BBA) Gen. Subj..

[13] Barreteau H., Richard E., Drouillard S., Samain E., Priem B. (2012). Production of intracellular heparosan and derived oligosaccharides by lyase expression in metabolically engineered *E. coli* K-12. Carbohydr. Res..

[14] Priem B., Peroux J., Colin-Morel P., Drouillard S., Fort S. (2017). Chemo-bacterial synthesis of conjugatable glycosaminoglycans. Carbohydr. Polym..

[15] Zhu H.-M., Sun B., Li Y.-J., Meng D.-H., Ju-Zheng S., Wang T.-T., Wang F., Sheng J.-Z. (2017). KfoA, the UDP-glucose-4-epimerase of *Escherichia coli* strain O5:K4:H4, shows preference for acetylated substrates. Appl. Microbiol. Biotechnol..

[16] Blumenkrantz N., Asboe-Hansen G. (1973). New method for quantitative determination of uronic acids. Anal. Biochem..

[17] Kidby D., Davidson D. (1973). A convenient ferricyanide estimation of reducing sugars in the nanomole range. Anal. Biochem..

[18] Stevenson G., Andrianopoulos K., Hobbs M., Reeves P.R. (1996). Organization of the *Escherichia coli* K-12 gene cluster responsible for production of the extracellular polysaccharide colanic acid. J. Bacteriol..

[19] Yavuz E., Drouillard S., Samain E., Roberts I., Priem B. (2007). Glucuronylation in *Escherichia coli* for the bacterial synthesis of the carbohydrate moiety of nonsulfated HNK-1. Glycobiology.

[20] Bastide L., Priem B., Fort S. (2011). Chemo-bacterial synthesis and immunoreactivity of a brain HNK-1 analogue. Carbohydr. Res..

[21] Studier F.W., Daegelen P., Lenski R.E., Maslov S., Kim J.F. (2009). Understanding the differences between genome sequences of *Escherichia coli* B Strains REL606 and BL21(DE3) and comparison of the *E. coli* B and K-12 genomes. J. Mol. Biol..

[22] Rye C.S., Withers S.G. (2002). Elucidation of the Mechanism of Polysaccharide Cleavage by Chondroitin AC Lyase from *Flavobacterium heparinum*. J. Am. Chem. Soc..

[23] Kakizaki I., Ibori N., Kojima K., Yamaguchi M., Endo M. (2010). Mechanism for the hydrolysis of hyaluronan oligosaccharides by bovine testicular hyaluronidase. FEBS J..

[24] Wolf S., Warnecke S., Ehrit J., Freiberger F., Gerardy-Schahn R., Meier C. (2012). Chemical synthesis and enzymatic testing of CMP-sialic acid derivatives. ChemBioChem.

[25] Pouilly S., Bourgeaux V., Piller F., Piller V. (2012). Evaluation of analogues of GalNAc as substrates for enzymes of the mammalian GalNAc salvage pathway. ACS Chem. Biol..

[26] Lane R.S., Ange K.S., Zolghadr B., Liu X., Schäffer C., Linhardt R.J., DeAngelis P.L. (2017). Expanding glycosaminoglycan chemical space: Towards the creation of sulfated analogs, novel polymers and chimeric constructs. Glycobiology.

[27] Gagnon S.M.L., Meloncelli P.J., Zheng R.B., Haji-Ghassemi O., Johal A.R., Borisova S.N., Lowary T.L., Evans S.V. (2015). High resolution structures of the human ABO(H) blood group enzymes in complex with donor analogs reveal that the enzymes utilize multiple donor conformations to bind substrates in a stepwise manner. J. Biol. Chem..

[28] Fort S., Birikaki L., Dubois M.-P., Antoine T., Samain E., Driguez H. (2005). Biosynthesis of conjugatable saccharidic moieties of GM2 and GM3 gangliosides by engineered *E. coli*. Chem. Commun..

