# Supplementary material for: Misincorporation of Galactose by Chondroitin Synthase of Escherichia coli K4: From Traces to Synthesis of Chondbiuronan, a Novel Chondroitin-Like Polysaccharide
Source: Biomolecules. 2020 Dec 12;10(12):1667. doi: 10.3390/biom10121667 (PMC7764085; doi:10.3390/biom10121667)
Supplement: Supplementary file 1 [file biomolecules-10-01667-s001.pdf]

biomolecules

Supplementary Materials

Misincorporation of Galactose by Chondroitin Synthase of Escherichia Coli K4: From Traces to Synthesis of Chondbiuronan, a Novel Chondroitin-like Polysaccharide

Mélanie Leroux [1,2], Julie Michaud [2], Eric Bayma [2], Sylvie Armand [2], Sophie Drouillard [2] and Bernard Priem [2,*]

[1] HTL biotechnology, 35133 Javene, France; mleroux@htlbiotech.com

[2] University Grenoble Alpes, CNRS, CERMAV, 38000 Grenoble, France; a.julie.michaud@gmail.com (J.M.); eric.bayma@cermav.cnrs.fr (E.B.); Sylvie.Armand@cermav.cnrs.fr (S.A.); sophie.drouillard@cermav.cnrs.fr (S.D.)

* Correspondence: bernard.priem@cermav.cnrs.fr

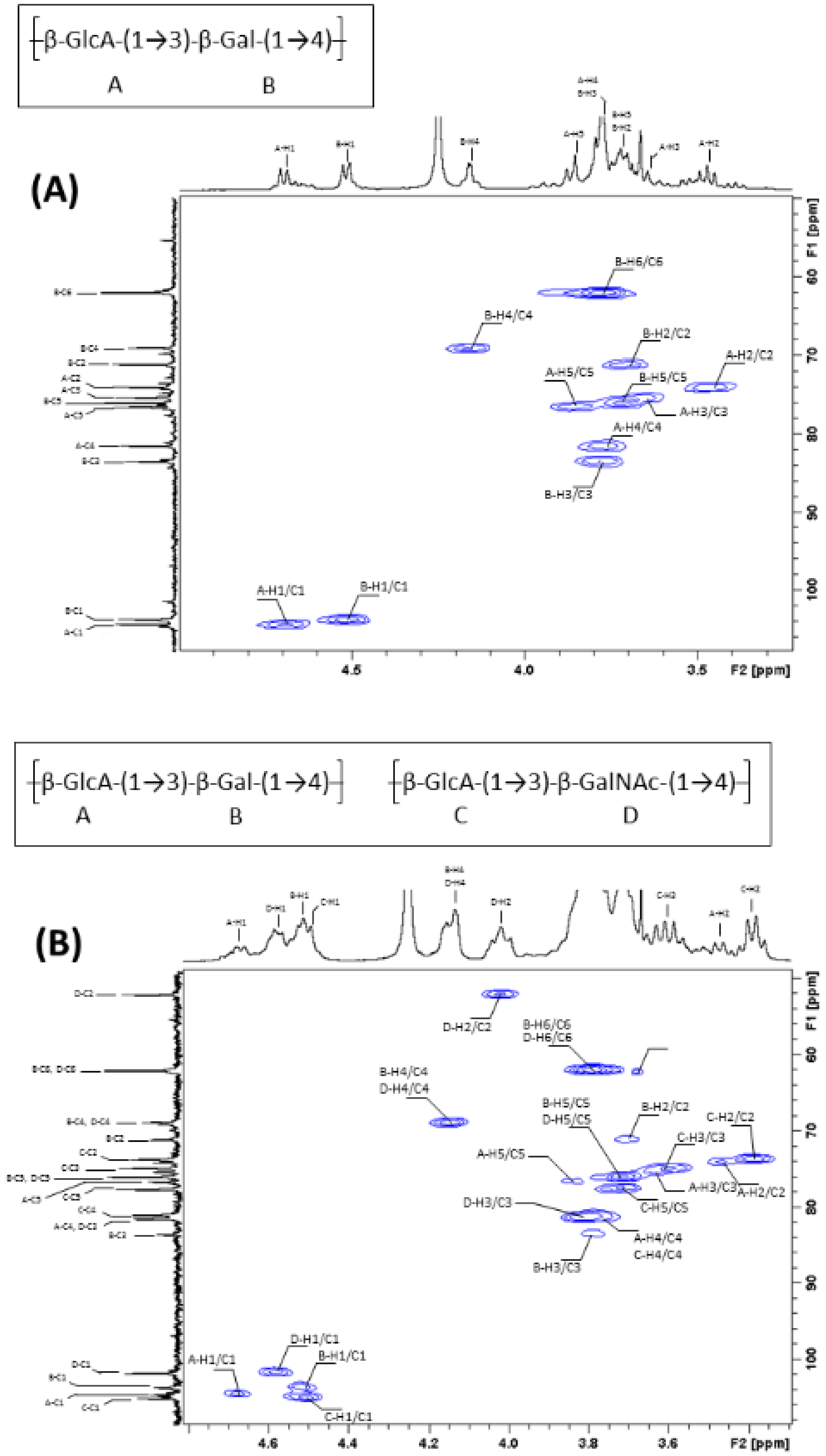

Figure S1. HSQC spectra of chondbiuronan (**A**) and chondroitin (**B**) obtained using 2% arabinose induction. Inset: chemical structure of the polysaccharides.

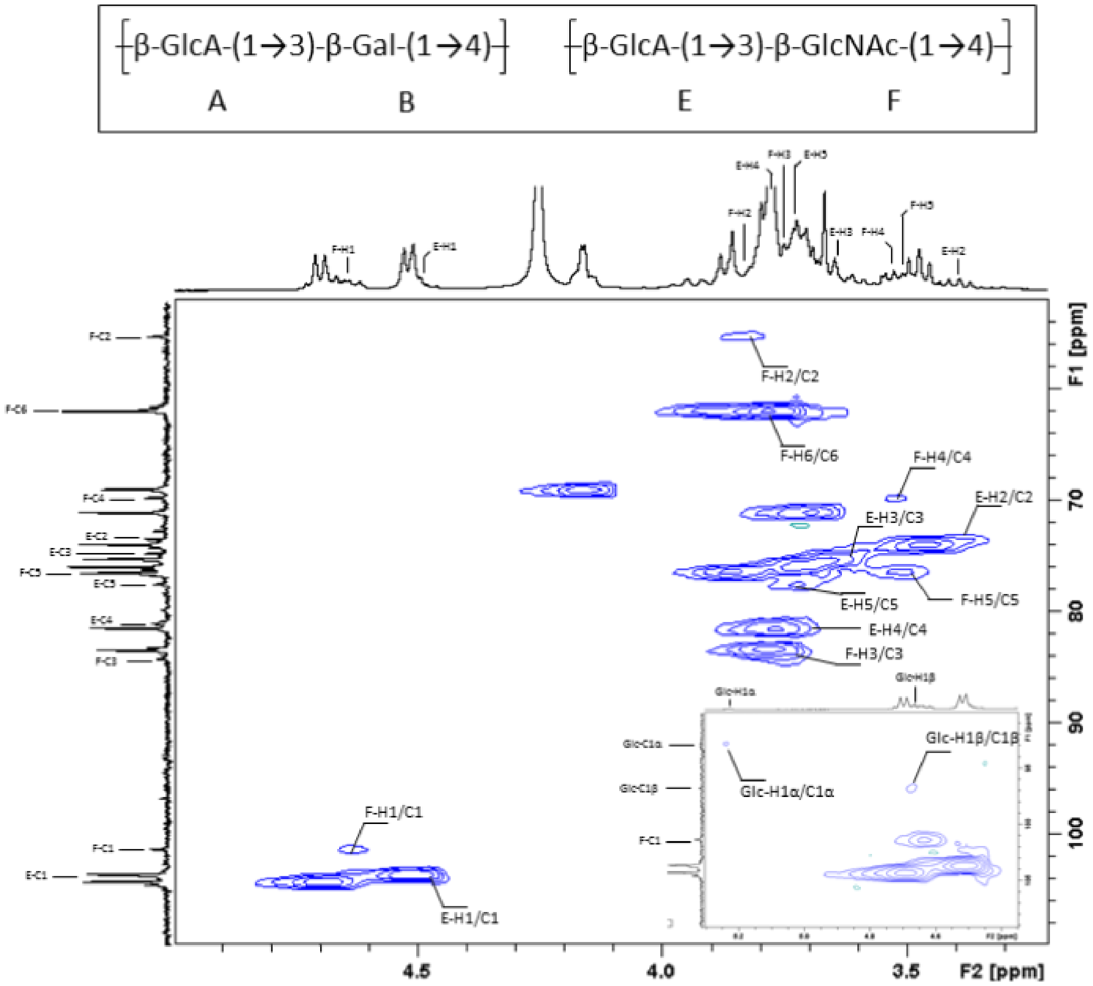

Sugar Residue		1	2	3	4	5	6 (6a,6b)	NHAc (CH3,CO)
Chondbiuronan EcDGCo								
E →4)-β-D-GlcA-(1→	^{1}H	4.50	3.39	3.64	3.78	3.73	no	
	^{13}C	103.65	73.44	74.77	81.06	77.53	175.11	
F →3)-β-D-GlcNAc-(1→	^{1}H	4.63	3.84	3.78	3.52	3.52	3.68, 3.93	2.04
	^{13}C	101.31	55.24	84.21	69.74	76.51	61.79	23.45, 175.67
→4)-α/β-D-Glc	^{1}H	5.23/4.67	3.59/3.30	nd	nd	nd	nd	
	^{13}C	92.7/96.8	nd	nd	nd	nd	nd	

Figure S2. HSQC spectrum of chondbiuronan showing the presence of Glc and GlcNAc incorporated in the polymer. Inset: chemical structure of the polysaccharides.

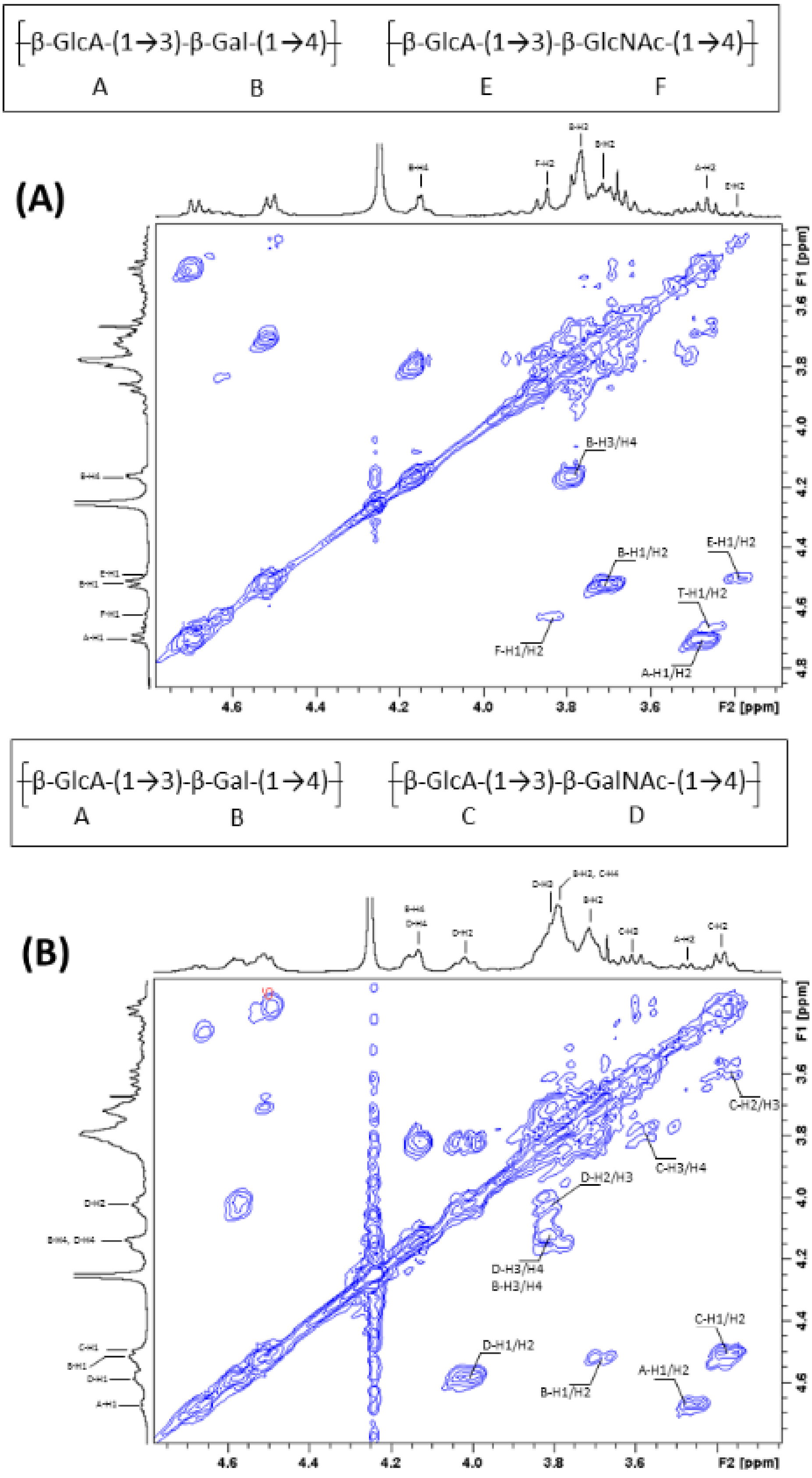

Figure S3. COSY spectra of chondbiuronan (**A**) and chondroitin (**B**) obtained using 2% arabinose induction. Inset: chemical structure of the polysaccharides.

Table S1. Genes, plasmids and *E. coli* strains used in this study.

Genes	Description	Ref. or source
GlcAT-P	Mouse glucuronyltransferase	GI 568960207
kfiD	UDP-glucose-6-dehydrogenase from *E. coli* K5	GI 735963
kfoA	UDP-GlcNAc 4-epimerase from *E. coli* K4	GI 21326786
kfoC	Chondroitin synthase from *E. coli* K4	GI 21326784
**Plasmids**		
pBBR1MCS3	Cloning vector, Tet, Plac promoter, bhr replicon	Kovach et al., 1995
pBAD33	Cloning vector, Cm, ParaBAD promoter, pSC101 replicon	Guzman, 1995
pBS	pBluescript II KS, Amp, colE1 replicon	Alting-Mees and Short, 1989
pBBR-glcATP-kfiD	pBBR1MCS3 expressing kfiD and GlcAT-P	Priem et al., 2017
pBS-kfoC	pBS expressing kfoC	Priem et al., 2017
pBAD-kfoA	pBAD33 expressing kfoA	This work
***E. coli* strains**		
**DH1**	F-, endA1, gyrA96, thi-1, hsdR17(rK-,mK+), supE44, relA1	DSMZ
**DJ**	Strain DH1 lacA lacZ wcaJ	Yavuz et al., 2008
**EcDGCø**	Strain DJ, pBBR-glcAT-kfiD; pBS-kfoC	This work
**EcDGCA**	Strain DJ, pBBR-glcAT- kfiD; pBS-kfoC; pBAD-kfoA	This work
